# Should Advanced Friedreich’s Ataxia Be a Contraindication for Heart Transplantation? A Case Report of a Successful Procedure in a 58-Year-Old Patient

**DOI:** 10.3390/jcdd9030080

**Published:** 2022-03-09

**Authors:** María Jesús Valero, Jose L. Muñoz-Blanco, Alejandro Garrido Sanchez, Gregorio Cuerpo, Javier Castrodeza, Paula Navas, Iago Sousa, Adolfo Villa, Francisco Fernández-Avilés, Manuel Martínez-Sellés

**Affiliations:** 1 Department of Cardiology, Hospital Universitario Gregorio Marañón, CIBERCV, 28007 Madrid, Spain; mariajesus.valero@salud.madrid.org (M.J.V.); jcastrodeza5@gmail.com (J.C.); paulanavastejedor@gmail.com (P.N.); iagosousa1979@gmail.com (I.S.); adolfovilla74@gmail.com (A.V.); ffaviles@gmail.com (F.F.-A.); 2 ALS-Neuromuscular Unit, Department of Neurology, Hospital General Universitario Gregorio Marañón, 28007 Madrid, Spain; joseluis.munoz@madrid.org; 3 Cardiac Surgery Postoperative Care Unit, Department of Anestesiology, Hospital General Universitario Gregorio Marañón, 28007 Madrid, Spain; alegasan@hotmail.com; 4 Department of Cardiac Surgery, Hospital General Universitario Gregorio Marañón, 28007 Madrid, Spain; grepa_genf@yahoo.es; 5 Department of Medicine, Universidad Complutense, 28040 Madrid, Spain; 6 Department of Medical Sciences, Universidad Europea, 28670 Madrid, Spain

**Keywords:** Friedreich’s ataxia, heart transplantation, ethics, prognosis, neuromuscular disease

## Abstract

The information on heart transplantation (HT) in patients with Friedreich’s Ataxia (FA) is scarce, and the few published case reports are limited to young patients with mild neurological manifestations. We present the case of a 58-year-old patient with advanced FA (Scale for the Assessment and Rating of Ataxia [SARA] score 30/40), wheelchair-bound for the last 16 years and had urinary incontinence, dysarthria, and neurosensorial deafness. The patient was admitted for a refractory arrhythmic storm and had previous hypertrophic cardiomyopathy that evolved to dilated cardiomyopathy with severely reduced left ventricular ejection fraction and recurrent ventricular arrhythmias. A multidisciplinary team discussed the HT option. The patient was aware of the risks and benefits and considered worthy of the intervention, so he was listed for HT. After a successful surgical intervention, the patient had a long postoperative stay in ICU. He required a high dose of vasopressors, underwent hemofiltration for one month, suffered critical illness myopathy, had several respiratory infections and delayed tracheal extubation. Two and a half months after HT and almost five months at the hospital, the patient was successfully discharged. FA patients with severe heart conditions should be carefully evaluated by a multidisciplinary team to decide the candidacy for HT.

## 1. Introduction

Neuromuscular diseases might be considered a contraindication for heart transplantation (HT). The information of HT in patients with Friedreich’s Ataxia (FA) is scarce, and the few published case reports are limited to young patients with mild neurological manifestations.

## 2. Detailed Case Description

We present the case of a 58-year-old patient, diagnosed with FA at the age of 33, with a confirmed genetic diagnosis and over 500 repetitions of the GAA triplet in both alleles of the first intron of the frataxin gene. He was attached to a wheelchair at the age of 42, with dysarthria, mild neurosensorial deafness, and a permanent urinary catheter due to incontinence and used a nasal continuous positive airway pressure due to obstructive sleep apnea syndrome. His Scale for the Assessment and Rating of Ataxia (SARA) [1] score was 30/40, and he had good social support and lived with his wife and daughter, the main caregivers. Heart disease started with left ventricular hypertrophy that evolved to dilated myocardiopathy with severely reduced left ventricular ejection fraction (20%), severe mitral regurgitation and recurrent episodes of supraventricular and ventricular arrhythmias. Three years before, sustained ventricular tachycardia was treated with an implantable cardioverter defibrillator and successful epicardial ablation. Medical treatment included betablockers, amiodarone, angiotensin neprilysin inhibitors, warfarin, and aldosterone receptor antagonists. During the last year, his cardiac condition was stable, without hospital admissions.

He was admitted to the hospital due to a ventricular arrhythmic storm. Despite intubation, deep sedation, intravenous amiodarone, and endocardial ablation, recurrent ventricular tachycardias persisted. A multidisciplinary group—that included cardiologists, neurologists, anesthesiologists, cardiac surgeons, and social workers—discussed the case and presented the different options to the patient and his family. The patient was included in HT regional priority waiting list and was successfully weaned off mechanical ventilation. After the association of mexiletine, ventricular arrhythmias frequency decreased, but severe progressive heart failure persisted. Two months after admission, HT was performed, with induction therapy with basiliximab and standard immunosuppression with mycophenolate, prednisone, and tacrolimus. High dose vasopressors were needed due to vasoplegic syndrome. Acute kidney failure required continuous and intermittent hemofiltration for 25 days. Tracheal extubation was achieved 20 days after HT. Non-invasive ventilation was prescribed intermittently, alternating with a high flow nasal cannula to prevent atelectasis. Furthermore, a specific respiratory physiotherapy plan was implemented to improve respiratory drive and secretions clearance. The patient received professional psychological support during the whole postoperative, recovered from upper limb myopathy, and his functional situation at discharge was similar to the one present before admission. He required nasogastric tube feeding with gastrostomy tube insertion, withdrawn five days before discharge. Almost 5 months after admission and 2 and a half months after HT, the patient was discharged with a normal cardiac function and a stable neurological situation during follow-up.

## 3. Discussion

FA is the most common inherited ataxia, with a prevalence of 1–5/100,000 [2]. It is mostly caused by a homozygous GAA triplet repeat expansion in the first intron of the frataxin gene with recessive inheritance. Age of disease onset and severity of cardiomyopathy are inversely correlated with the number of repetitions of this triplet and with a more rapid progression [3]. FA phenotype typically involves the nervous system, musculoskeletal, myocardium, and endocrine pancreas. The symptoms usually appear during adolescence, and most patients are wheelchair-bound in the third decade. Clinical manifestations start affecting the gait and include dysarthria, pyramidal weakness, and dysphagia [4]. Sensorineural deafness can be present, such as subtle cognitive impairment. Cardiomyopathy is the main cause of death [5,6] related to left ventricular hypertrophy, although a small group of patients may progress to a dilated cardiomyopathy with reduced ejection fraction. Supraventricular and ventricular arrhythmias and heart failure are common, and the mean age at death is 36.5 years [7]. Atypical phenotypes have been described as late-onset FA and very late-onset FA, when first symptoms appear after the age of 25 and 40, respectively [4], with milder symptoms and a slower progression. Current management is limited to symptoms treatment, and patients should be referred to genetic counselling for inheritance implications. Physiotherapy and rehabilitation may counteract the effects of progressive physical deterioration. Other important areas that need to be supported are speech therapy, palliative care, and medical assistance (cardiology, sleep physician, urology). Cardiovascular management should take into consideration symptoms, left ventricular outflow gradient, and sudden death risk [7]. Gene therapy could be a promising treatment, but it is not still a reality [8]. Patients with severe cardiac manifestations have a poor prognosis. The main mortality predictors are the length of GAA in the Frataxin gene and the degree of left ventricular hypertrophy and systolic dysfunction [5].

Two case series have reported FA patients treated with HT. McCormick et al. [9] reported three cases with favorable long-term follow up but, in one of them, AF diagnosis was only performed after HT. In the other two patients, HT was performed at the age of 37. Segovia et al. reported three cases with favorable follow up [9], all in their twenties at the time of HT, one wheelchair-bound. Two were listed for emergency HT due to cardiac arrest. Our patient and five previous case reports [10,11,12,13,14] add to the list of 12 successful HT in patients with FA (Table 1).

In previous cases, HT was mainly performed at a young age and FA was previously undiagnosed or mild. Interestingly, neurological evolution after HT seems to be worse in younger patients. Our case provides novelty due to several reasons: (1) our patient is the oldest FA patient to receive HT; (2) this is the only case in which HT was performed due to uncontrolled ventricular arrhythmias; (3) our patient was wheelchair-bound for the last 16 years. Only a previous case of a wheelchair-bound patient has been published, a young patient recently wheelchair-bound. Late-onset FA usually has a slower progression, and, in our patient, the slow progression and neurological stability during the last 20 led us to expect longer survival than the mean life expectancy of FA patients [16]. As the expected life due to neuromuscular affection exceeded the medium graft survival time, HT seemed to be the best treatment option for his cardiomyopathy. In any case, the high impact of Friedreich ataxia on quality of life is well known and should be taken into consideration. This impact includes motor disability but is also present on non-motor dimensions and depressive symptomatology [17].

Our case suggests the need to consider patients with FA and advanced HF as candidates for HT. The inclusion and exclusion criteria used for these patients regarding heart failure should be similar to the ones used in other patients. However, a multidisciplinary evaluation is essential. Patients with a predicted poor neurological evolution should not be considered for HT and should receive the available treatments for advanced heart failure.

## 4. Conclusions

We conclude that HT is a safe option for end-stage heart disease in selected patients with FA. A multidisciplinary team should assess utility, justice, quality of life, and life expectancy. Patient involvement in the decision is essential.

## Figures and Tables

**Table 1:** Cases of Freidereich’s ataxia (FA) patients treated with heart transplant (HT).

	Age at HT (Years)	Sex	FA Diagnosed before HT
McCormick et al. [8]	5	Male	No
	37	Male	Yes
	37	Female	Yes
Segovia et al. [9]	26	Male	Yes
	24	Male	Yes
	26	Male	Yes
Ivak et al. [15]	23	Female	Yes
Leonard et al. [10]	4	Male	No
Sedlak et al. [11]	34	Male	Yes
Silva et al. [12]	22	Male	No
Yoon et al. [13]	14	Male	Yes
Our case	58	Male	Yes
